# Efficacy and Safety of Jiawei Suanzaoren Decoction Combined with Lorazepam for Chronic Insomnia: A Parallel-Group Randomized Controlled Trial

**DOI:** 10.1155/2020/3450989

**Published:** 2020-02-08

**Authors:** Ming-Fen Song, Li-Qiong Chen, Qiong-Yan Shao, Lin-Lin Hu, Wen-Juan Liu, Yong-Hua Zhang

**Affiliations:** ^1^Molecular Biology Laboratory, Hangzhou Seventh People's Hospital, Hangzhou, Zhejiang, China; ^2^Hangzhou Hospital of Traditional Chinese Medicine, Guang Xing Hospital Affiliated to Zhejiang Chinese Medical University, Zhejiang Chinese Medical University, Hangzhou, Zhejiang, China; ^3^Department of Psychosomatic Disorders, Hangzhou Seventh People's Hospital, Hangzhou, Zhejiang, China; ^4^Department of Internal Neurology, Hangzhou Hospital of Traditional Chinese Medicine, Hangzhou, Zhejiang, China

## Abstract

**Background:**

Chronic insomnia is a major public health problem, but there are limited effective therapies. Jiawei Suanzaoren Decoction (JW-SZRD) has been used as an alternative option for treating insomnia. This study aimed to investigate the long-term efficacy and safety of JW-SZRD in combination with lorazepam for chronic insomnia.

**Methods:**

A total of 207 participants were analyzed in this study. The treatment group (TG) received JW-SZRD and lorazepam orally, and the control group (CG) received lorazepam alone. The Insomnia Severity Index (ISI), the Self-Rating Depression Scale (SDS), the Self-Rating Anxiety Scale (SAS), and the Somatic Self-rating Scale (SSS) were evaluated at baseline, weeks 4, 8, and 12. The MOS 36-item Short Form Health Survey (SF-36) was assessed at baseline and week 12. Adverse effects (AEs) were evaluated by the Treatment Emergent Symptom Scale (TESS).

**Results:**

Both TG and CG showed obvious improvements in the sleep onset latency (SOL) (*P*=0.001 and 0.005) and total sleep time (TST) (*P*=0.001 and 0.005) and total sleep time (TST) (*P*=0.001 and 0.005) and total sleep time (TST) (*P*=0.001 and 0.005) and total sleep time (TST) (*P*=0.001 and 0.005) and total sleep time (TST) (*P*=0.001 and 0.005) and total sleep time (TST) (*d* = 1.28). The ISI reduction rate in TG was higher than that in CG at weeks 4, 8, and 12 (*P*=0.001 and 0.005) and total sleep time (TST) (*P*=0.001 and 0.005) and total sleep time (TST) (*P*=0.001 and 0.005) and total sleep time (TST) (*P*=0.001 and 0.005) and total sleep time (TST) (*P*=0.001 and 0.005) and total sleep time (TST) (*P*=0.001 and 0.005) and total sleep time (TST) (

**Conclusion:**

The combination of JW-SZRD with lorazepam can significantly improve sleep quality with fewer AEs. It is an effective treatment and superior to lorazepam alone for chronic insomnia.

## 1. Introduction

Chronic insomnia, which is defined as the persistent difficulty in initiating sleep, maintaining sleep, nonrestorative sleep, or poor-quality sleep over a period of at least three months, has already become a common public health concern worldwide [1]. It was reported that nearly one-third of the general population had experienced insomnia symptoms, among which 10.8% met the criteria of insomnia disorder in DSM-5 [2] and approximately 7% were diagnosed as chronic insomnia disorder [3]. Insomnia can cause mental dysfunction and daytime sleepiness. In some cases, it can even result in physiological and psychological disorders, such as depression, anxiety, and perceived stress [4, 5].

Currently, pharmacological treatment has been widely used for patients with chronic insomnia. However, drugs are always considered to be associated with physical and psychological dependencies, impairment of cognitive function, daytime drowsiness and anxiety, rebound insomnia, and so on [6] although less and less adverse effects (AEs) would happen with the development of pharmaceutical technology [7].

Traditional Chinese Medicine (TCM) has a long history of thousands of years and is widely accepted for various disease treatment because of their obvious therapeutic efficacy, natural plant source and less AEs. Recently, some researchers have been trying to find out a useful herbal formula for the treatment of insomnia.

Suanzaoren decoction (SZRD) is a classic Chinese herbal formula with hypnotic, sedative, and anticonvulsant properties. Despite the apparent positive results [8, 9], there is still insufficient evidence to support its use for insomnia due to the poor methodological quality and the small number of trials of the previous studies [10, 11]. In addition, chronic insomnia often presents negative emotions such as anxiety and depression, which require additional pharmacological interventions because of the limited efficacy of SZRD alone. Zhizichi decoction (ZZCD), comprising of *Gardenia jasminoides fruit* and *fermented soybean*, is also a traditional Chinese formula that has been widely used for depression and insomnia treatment for more than two thousand years [12]. In recent years, a combined formula of SZRD and ZZCD (Jiawei Suanzaoren Decoction, JW-SZRD) has been proven to be effective for insomnia with yin-deficiency and fire-excess syndrome [13, 14]. Yin-deficiency and fire-excess syndrome usually shows palpitation, restlessness, headache, rapid pulse, hot flashes, and excessive sweating and is one of the common patterns of insomnia in clinical practice. However, no consistency was obtained in pattern-based TCM treatment of insomnia, and specific treatment principles underlying herb formula selection were seldom reported [15].

In our previous research, we have verified the short-term efficacy of JW-SZRD for insomnia patients with yin-deficiency and fire-excess syndrome [14]. However, the long-term effects of JW-SZRD for chronic insomnia remain unclear. In this study, we aimed to investigate the long-term efficacy and safety of JW-SZRD when used in combination with lorazepam for the treatment of chronic insomnia.

## 2. Materials and Methods

### 2.1. Patients

240 patients (154 females and 86 males) with chronic insomnia from Hangzhou Seventh People's Hospital between January 2014 and December 2017 were eligible to be recruited in this study. The inclusion criteria were as follows: (a) fulfilling the diagnosis of chronic insomnia according to the Diagnostic and Statistical Manual of Mental Disorders, Fifth Edition (DSM-5); (b) primary insomnia; (c) yin-deficiency and fire-excess syndrome, which was evaluated by a Chinese medicine practitioner and validated by Yong-Hua Zhang (a famous Chinese medicine practitioner in Zhejiang Province with more than 30 years of experience); (d) between 18 and 60 years old; and (e) an education level of junior high school or above to obtain a better compliance and reduce the drop-off rate. The exclusion criteria included the following: (a) secondary insomnia caused by other diseases such as psychotic disorders or somatic diseases; (b) women who were pregnant, lactating, or without contraception; (c) abnormal liver function; (d) working on night shifts; (e) tea-drinkers; (f) coffee-drinkers; (g) alcohol drinkers; (h) cigarette smokers.

**Figure 1:**
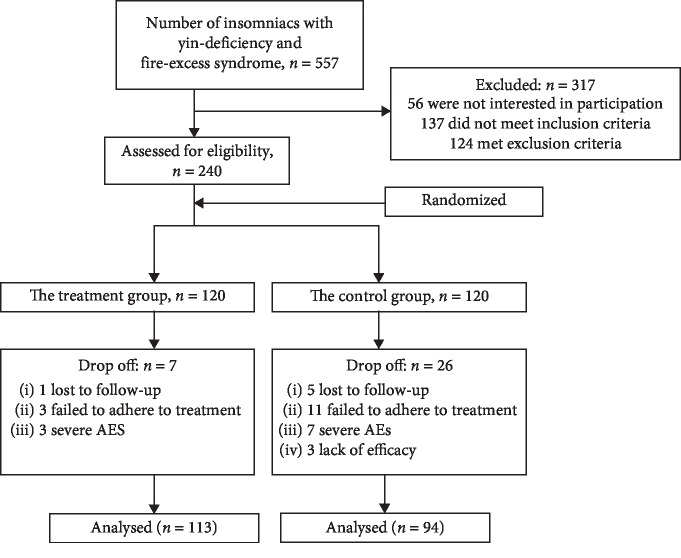
Flowchart of subject enrollment.

The study was approved by the Ethics Committee of Hangzhou Seventh People's Hospital (2014-001). All participants signed informed consent forms before participation. The flowchart of subject enrollment was shown in Figure 1.

### 2.2. Treatment

Using a permuted block randomization scheme [16], the eligible patients were randomized 1 : 1 into two groups: the treatment group (TG; *n* = 120) and the control group (CG; *n* = 120). Patients in TG were treated with JW-SZRD (150 ml each time, twice daily, half an hour after breakfast and dinner) and lorazepam (0.5 mg/day, half an hour after dinner) orally, while cases in CG were given lorazepam alone (0.5 mg/day, half an hour after dinner). For TG, the daily dosage of JW-SZRD was composed of *Suanzaoren* 15 g, *Fuling* 15 g, *Chuanxiong* 10 g, *Zhimu* 10 g, *Zhizi* 10 g, *Dandouchi* 10 g, and *Gancao* 6 g (See Table 1). All herbs were purchased from Medicinal Materials Co. Ltd. (Lin'an City, Zhejiang Province, China). These ingredients were mixed, decocted into a 300 ml solution, and vacuum-packed into two bags by the same company. Lorazepam (0.5 mg/tablet) was purchased from Hainan Daxiyan Pharmaceutical Co., Ltd., China. The treatment lasted for 12 weeks and the follow-up investigations were scheduled at baseline, weeks 4, 8, and 12. All the subjects were required no consumption of tea, coffee, alcohol, and cigarette during the whole study period to avoid their potential influences on the treatment efficacy.

Patients and investigators giving interventions were not masked to the treatment assignment. However, the outcome evaluators and statisticians were blinded to the group allocation.

During the whole study period, 7 cases quit in TG (1 lost to follow-up, 3 failed to adhere to treatment, 3 had serious adverse effects) and 26 cases withdrew in CG (5 lost to follow-up, 11 failed to adhere to treatment, 7 had severe adverse effects, 3 were lack of efficacy). Finally, 113 cases in TG and 94 cases in CG were analyzed.

### 2.3. Efficacy Evaluation

The Insomnia Severity Index (ISI) was used for the primary outcome measurement [17, 18]. The Insomnia Severity Index (ISI) is a self-reported questionnaire and its Chinese version was tested to be valid for assessing insomnia [19], including the current severity of insomnia symptoms, sleep dissatisfaction, daytime impacts, and distress about sleep difficulties [20].

ISI evaluations were performed at baseline and weeks 4, 8, and 12. All participants were trained to maintain daily sleep diaries for a 7-day baseline period and the 12-week treatment period. The parameters in the diaries included bedtime, arising time, medication intake, number and duration of awakenings, and morning alertness or sluggishness. Based on these parameters, the following outcomes were calculated: sleep onset latency (SOL), total sleep time (TST), and the number of night-time awakenings (NNTA).

The secondary outcomes included anxiety, depression, and health-related quality of life. Anxiety was evaluated according to the 20-item Self-Rating Anxiety Scale (SAS), and depression was assessed by using the 20-item Self-Rating Depression Scale (SDS). In addition, Somatic Self-rating Scale (SSS) was also reported by patients. These scales were performed at baseline, weeks 4, 8, and 12. Health-related quality of life was measured based on the 36-item Short Form Health Survey (SF-36) at baseline and week 12 [21].

### 2.4. Assessment of Adverse Effects (AEs)

The Treatment Emergent Symptom Scale (TESS) was used to evaluate the AEs as mentioned in other papers [22, 23], including behavioral toxicity, laboratory examination abnormalities, nervous system, autonomic nervous system, cardiovascular system, body weight, headache, and appetite. The value for each item ranged from 0 to 4: zero meant no AEs, one represented mild AEs, two showed moderate AEs, three exhibited severe AEs, and four indicated very severe AEs.

### 2.5. Statistical Analysis

SPSS 19.0 software (IBM Corp., Armonk, NY, USA) was used for statistical analyses. *χ*^*2*^ Test was conducted for the categorical data and Student's *t*-test was analyzed for the continuous variables between two groups. The analysis of repeated measurement ANOVA was performed to compare the differences of ISI, SAS, and SDS scores at baseline, weeks 4, 8, and 12. Paired *t*-test was run to compare the score changes of SF-36 between baseline and week 12. Probability (*P*) values < 0.05 were considered statistically significant. Cohen's d values were calculated for the evaluations of effect sizes.

## 3. Results

### 3.1. Demographic Characteristics

The demographic characteristics were summarized in Table 2. There was no significant difference between TG and CG (all *P* > 0.05).

### 3.2. Comparison of ISI Scores

Compared with baseline, both groups had significant changes in SOL and TST (all *P* < 0.01). Compared with CG, TG had significantly shorter SOL and obviously longer TST at weeks 8 and 12 (*P* < 0.05 or 0.01). However, there was no significant difference in NNTA between the two groups at all observation points (*P* > 0.05). Cohen's *d* was 1.28 for SOL, 0.16 for TST, and 0.23 for NNTA. Thus, shortening SOL of chronic insomnia was the main therapeutic action of JW-SZRD as shown in Table 3.

**Figure 2:**
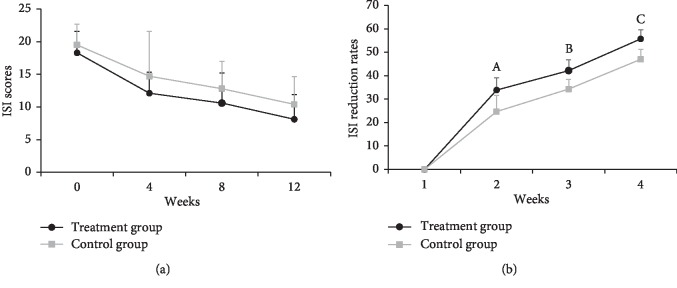
ISI score changes in both groups during the 12-week treatment. Compared to the control group, ^A^*t* = 2.69, *P*=0.008; ^B^*t* = 3.22, *P*=0.001; ^C^*t* = 3.35, *P*=0.001.

Following the 12-week treatment, the ISI scores were both reduced in TG and CG, but there was no significant difference between groups (*P* > 0.05). However, the ISI reduction rate in TG was higher than that in CG at weeks 4, 8, and 12 (all *P* < 0.01) as shown in Figure 2.

### 3.3. Comparison of SAS and SDS Scores

**Figure 3:**
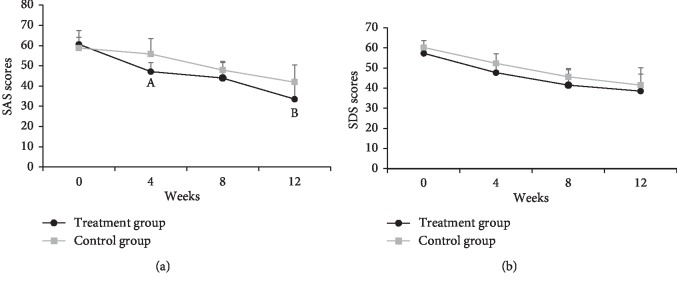
SAS and SDS score changes in both groups during the 12-week treatment. ^A^*t* = 3.81, *P*=0.0001; ^B^*t* = 2.73, *P*=0.007 when compared to the control group.

Patients in TG had lower SAS scores than those in CG at weeks 4 (Cohen's *d* = 0.53) and 12 (Cohen's *d* = 0.38) (both *P* < 0.01). There was no significant difference in SDS scores between two groups at all observation points (all *P* > 0.05) Shown in Figure 3.

### 3.4. Comparison of SSS Scores

As not all patients had somatic symptoms, we compared the case numbers instead of the mean value between the two groups at each evaluation point. At baseline, the case numbers of all symptoms were not significantly different between the two groups (*P* > 0.05). After treatment, TG had fewer subjects who suffered from somatic symptoms of dizziness and headache, chest discomfort and palpitation, loss of appetite, constipation, throat discomfort, hot flashes, and night sweating as compared with those of CG at weeks 4, 8, or 12 (*P* < 0.05 or 0.01) (as shown in Table 4).

### 3.5. Comparison of SF-36 Scores

Both TG and CG had significant changes in all eight dimension scores of SF-36 at week 12 as compared to baseline (*P* < 0.05 or 0.01). When compared between groups, there was no significant difference in all SF-36 dimension scores at baseline (all *P* > 0.05). However, after a 12-week treatment, patients in TG had significantly higher scores in physical functioning, role-physical, general health, vitality, social functioning, and role-emotional, but obviously lower values in body pain than those in CG (*P* < 0.05 or 0.01). The effect sizes were medium in role-physical (0.47), vitality (0.58), and role-emotional (0.60), but small in the other dimensions (Cohen's *d* values were close to 0.2), see Table 5.

### 3.6. Comparison of Compliance

The compliance rate in TG (94.12%, 113/120) was significantly higher than that in CG (78.33%, 94/120) (*χ*^*2*^ = 12.68, *P*=0.0001).

### 3.7. Comparison of Adverse Effects (AEs)

A total of 10 participants (3 in TG, and 7 in CG) withdrew from the study due to serious AEs. The subjects who completed our study had mild or moderate AEs as follows: constipation, loss of appetite, dizziness, headache, abnormal liver function, and sexual dysfunction. The incidence rates of AEs in TG were significantly lower than those in CG (*P* < 0.05 or 0.01) as shown in Table 6.

## 4. Discussion

In this study, we conducted a long-term investigation via a 12-week administration of concurrent use of JW-SZRD and lorazepam to the chronic insomnia patients and compared the treatment efficacy and safety with lorazepam alone.

Suanzaoren (*Ziziphi Spinosae Semen*) is the most commonly used single herb to treat insomnia, anxiety, and night sweating [11]. Recent pharmacological studies showed that Suanzaoren (*Ziziphi Spinosae Semen*) had multiple active constituents and exerted various pharmacological effects, which included antihyperlipidemia, immunopotentiation, and anxiolytic effects [24]. Although a single herb can have beneficial effects in the prevention and treatment of sleep disorders, it was believed that two or more compounds may have additive or synergistic effects when used together. Our previous study confirmed the short-term efficacy of JW-SZRD for insomniacs with anxiety and found that the administration of JW-SZRD for four weeks had a pronounced improvement in sleep quality and a remarkable alleviation of anxiety state [14].

However, JW-SZRD alone was not always effective in treating chronic insomnia. Based on our clinical experience, about one-third of the chronic insomniacs had no response to JW-SZRD and the efficacy in responders may even differ during the long-term treatment (data not provided). Previous studies reported that Chinese herbal formula could be coadministered as an adjuvant agent with hypnotic drugs to treat patients with insomnia [25]. Thus, we designed the current 12-week, parallel-group, randomized controlled trial to further investigate the long-term efficacy and safety of JW-SZRD combined with lorazepam for patients with chronic insomnia. Our findings indicated that the combination of JW-SZRD with lorazepam could alleviate insomnia (especially shorten the SOL), somatic, and anxiety symptoms better than lorazepam alone. Meanwhile, we also observed fewer incidence of AEs and better compliance in the combined group than those in the lorazepam group.

The combination of JW-SZRD and lorazepam showed more effectiveness than lorazepam alone, but the underlying mechanism is still unknown. We speculated that JW-SZRD may participate in regulating some neurotransmitter systems and thus have a synergistic interaction with benzodiazepines based on the evidence as follows. First, the therapeutic effects of SZRD may mediate through serotonergic activation. The water extract of Suanzaoren showed an ability of binding 5-HT1A and 5-HT2 receptors [26], and SZRD-induced nonrapid eye movement sleep (NREMS) could be blocked by administration of either 5-HT1A antagonist (NAN-190), 5-HT2 antagonist (ketanserin) or 5-HT3 antagonist (3-(4-Allylpiperazin-1-yl)-2-quinoxalinecarbonitrile) [27]. Second, SZRD may also affect the activity of the GABAergic system [8]. Yi et al. reported that SZRD exerted treatment effects through GABA_A_ receptor, but not GABA_B_ receptor due to the fact that intracerebroventricular (ICV) administration of GABA_A_ receptor antagonist, bicuculline, significantly blocked SZRD-induced enhancement in NREMS, but GABA_B_ receptor antagonist, 2-hydroxysaclofen, had no effect [28]. Third, the components of amino acid and fatty acid in SZRD would also be in response to the treatment effect through the immune and nervous system [29]. Fourth, JW-SZRD may also inhibit the hyperactivity of the HPA axis based on our clinical observation. However, relevant evidence is still lacking. Although ZZCD is also a classical formula and is widely used for insomnia in Chinese clinical applications, its mechanism with/without SZRT has seldom been studied. Further studies are needed to demonstrate the possible mechanism when SZRD and ZZCD (JW-SZRD) are used together for insomnia treatment.

In terms of AEs, gastrointestinal reactions such as nausea, diarrhea, constipation, and loss of appetite were the main effects induced by JW-SZRD. Of course, abnormal liver function or sexual dysfunction were also common [30]. These AEs were also observed when lorazepam alone was used. Instead of increasing the incidence rate of AEs when they were used together, in this study, we found that JW-SZRD could diminish the occurrence of AEs caused by lorazepam. It may be associated with the health-promoting and immunoprotective functions of JW-SZRD due to its abundant constituents [31, 32].

There are several limitations in our study. First, the design was not double-blinded as the traditional Chinese medicine decoction has unique features such as the special odor and taste, and the participants usually knew what they were receiving. Second, according to the clinical practice, the scales selected for the outcome assessments were self-reported, which may lead to subjective bias. Third, we have not performed the experiments to illuminate the exact mechanism of JW-SZRD in the abilities of insomnia treatment and AEs reduction. Thus, further investigations with a double-blinded design, objective indicators, and the mechanism of JW-SZRD are needed.

## 5. Conclusion

The concurrent use of JW-SZRD and lorazepam is an effective treatment for chronic insomnia, which can significantly improve sleep quality with less AEs and is superior to lorazepam alone.

## Figures and Tables

**Table 1:** The ingredients of JW-SZRD.

Pharmaceutical name	Chinese name	Latin botanical name	Proportion (%)
*Semen Ziziphi Spinosae*	Suanzaoren	*Ziziphus jujuba Mill*. var. *Spinosa*	19.74
*Sclerotium Poriae Cocos*	Fuling	*Poria cocos (Schw*.*) Wolf*	19.74
*Radix Ligustici Chuanxiong*	Chuanxiong	*Ligusticum chuanxiong Hort*.	13.16
*Rhizoma Anemarrhena*	Zhimu	*Anemarrhena asphodeloides Bge*.	13.16
*Gardenia Jasminoides fruit*	Zhizi	*Gardenia jasminoides Ellis*	13.16
*Fermented Soybean*	Dandouchi	*Semen Sojae Praepatum*	13.16
*Radix Glycyrrhizae*	Gancao	*Glycyrrhiza uralensis Fisch*.	7.88

**Table 2:** Demographic characteristics of the participants.

Characteristics		TG	CG	*t/χ* ^*2*^ value	*P* value
Gender (*n*)	Male	39	31	0.05	0.82
	Female	74	63				
Age (years)		44.0 ± 10.45	39.3 ± 9.36	1.43	0.15
Education (years)	≥8	38	33	0.83	0.84
	≥11	45	41				
	≥14	25	17		
	≥16	5	3		
Tea drinking		0	0	—	—
Coffee drinking		0	0	—	—
Alcohol drinking		0	0	—	—
Cigarette smoking		0	0	—	—

TG: the treatment group; CG: the control group.

**Table 3:** Sleep diary parameters in both groups.

Group	Time	SOL (min)	TST (min)	NNTA (*n*)
TG	Baseline	67.4 ± 26.27	192.2 ± 54.14	3.3 ± 1.27
	4-week	38.4 ± 10.86^*∗∗*^	312.5 ± 47.59^*∗∗*^	2.8 ± 1.45
	8-week	28.2 ± 7.05^*∗∗*#^	329.5 ± 41.46^*∗*#^	2.6 ± 1.16
	12-week	22.0 ± 4.14^*∗*##^	378.8 ± 53.03^*∗*#^	2.3 ± 1.15
	*F* value of ANOVA	5.34	7.49	2.03
	*P* value	0.001	0.0001	0.11
CG	Baseline	75.6 ± 23.27	182.7 ± 51.04	3.9 ± 1.83
	4-week	45.9 ± 13.93^*∗∗*^	223.9 ± 55.6^*∗∗*^	3.2 ± 1.68
	8-week	36.5 ± 13.19^*∗∗*^	337.2 ± 47.88^*∗∗*^	2.8 ± 1.13
	12-week	29.1 ± 6.67^*∗*^	368.7 ± 72.86^*∗*^	2.6 ± 1.41
	*F* value of ANOVA	4.28	5.72	1.67
	*P* value	0.005	0.001	0.17
Cohen's *d*		1.28	0.16	0.23

Data were expressed as mean ± SD. TG: the treatment group, CG: the control group. SOL: sleep onset latency; TST: total sleep time; NNTA: the number of night-time awakenings. ^*∗*^*P* < 0.05 and ^*∗∗*^*P* < 0.01 when compared with baseline in the same group. ^#^*P* < 0.05 and ^##^*P* < 0.01 when compared with CG at the same observation point.

**Table 4:** Somatic Self-rating Scale (SSS) in both groups.

Symptoms	Group	Baseline	Week 4	Week 8	Week 12
Dizziness and headache	TG	46	11	6	2
	CG	41	37	23	16
	*χ* ^*2*^ value	0.18	25.29	15.63	15.03
	*P* value	0.67	0.0001	0.0001	0.0001
Chest discomfort and palpitation	TG	92	57	23	3
	CG	83	57	31	17
	*χ* ^*2*^ value	1.86	2.16	4.24	14.00
	*P* value	0.17	0.14	0.04	0.0001
Loss of appetite	TG	23	18	6	2
	CG	18	23	17	8
	*χ* ^*2*^ value	0.05	2.36	8.48	5.07
	*P* value	0.83	0.13	0.004	0.02
Constipation	TG	11	16	8	4
	CG	12	34	32	27
	*χ* ^*2*^ value	0.48	13.57	23.93	25.56
	*P* value	0.49	0.0001	0.0001	0.0001
Throat discomfort	TG	21	15	11	3
	CG	16	14	14	11
	*χ* ^*2*^ value	0.09	0.11	1.29	6.66
	*P* value	0.77	0.74	0.26	0.01
Hot flashes and night sweating	TG	15	8	4	1^*∗∗*^
	CG	15	16	12	9
	*χ* ^*2*^ value	0.30	4.95	6.12	11.00
	*P* value	0.86	0.03	0.01	0.001

Data were shown in case numbers. TG: the treatment group, CG: the control group.

**Table 5:** SF-36 scores in two groups.

Dimensions	TG				CG				*t* value^∗^	*P* value	Cohen's *d*
	Baseline	Week 12	*t* value^*∗*^	*P* value	Baseline	Week 12	*t* value^#^	*P* value			
Physical functioning	83.0 ± 3.3	92.9 ± 2.6	2.65	0.01	82.7 ± 3.1	86.4 ± 2.4	1.38	0.17	2.68	0.008	0.37
Role-physical	44.1 ± 3.3	59.9 ± 3.6	2.61	0.01	43.0 ± 3.0	56.1 ± 4.7	2.17	0.03	3.36	0.0001	0.47
Body pain	19.5 ± 5.6	2.4 ± 5.0	3.78	0.0001	20.4 ± 3.6	11.5 ± 5.6	2.51	0.01	2.47	0.01	0.34
General health	35.7 ± 2.3	67 ± 3.9	3.97	0.0001	34.3 ± 2.6	55.9 ± 3.5	2.42	0.02	2.75	0.006	0.38
Vitality	31.6 ± 1.5	63 ± 2.7	2.84	0.01	31.6 ± 1.8	53.8 ± 2.5	4.06	0.0001	4.14	0.0001	0.58
Social functioning	41.6 ± 5.0	60 ± 6.1	4.21	0.0001	42.9 ± 5.7	55.1 ± 6.3	2.22	0.03	2.66	0.008	0.37
Role-emotional	53.7 ± 7.7	86 ± 9.2	2.29	0.02	56.4 ± 7.7	69.8 ± 9.3	2.64	0.01	4.31	0.0001	0.60
Mental health	42.2 ± 6.6	68 ± 7.1	2.55	0.01	45.2 ± 6.7	64.1 ± 6.9	3.88	0.0001	2.15	0.03	0.30

TG: the treatment group, CG: the control group. ^*∗*^Paired *t*-test between baseline and week 12 in the same group. ^#^Student's *t*-test for score-change comparison between the two groups.

**Table 6:** Adverse effects in both groups.

Groups	Constipation	Loss of appetite	Dizziness	Abnormal liver function	Sexual dysfunction	Others
TG	6	7	2	1	5	3
CG	33	19	13	6	12	10
*χ* ^*2*^	29.80	9.18	9.32	4.75	4.74	5.56
*P* value	0.0001	0.002	0.002	0.03	0.03	0.02

TG: the treatment group, CG: the control group.

## Data Availability

The data used to support the findings of this study are available from the corresponding author upon request.
